# *Babesia crassa*–Like Human Infection Indicating Need for Adapted PCR Diagnosis of Babesiosis, France

**DOI:** 10.3201/eid2802.211596

**Published:** 2022-02

**Authors:** Cécile Doderer-Lang, Denis Filisetti, Julie Badin, Charles Delale, Victoria Clavier, Julie Brunet, Chloé Gommenginger, Ahmed Abou-Bacar, Alexander W. Pfaff

**Affiliations:** Université de Strasbourg UR 7292, France (C. Doderer-Lang, D. Filisetti, J. Brunet, A. Abou-Bacar, A.W. Pfaff);; Hôpitaux Universitaires de Strasbourg, Strasbourg (D. Filisetti, J. Brunet, C. Gommenginger, A. Abou-Bacar, A.W. Pfaff);; Centre Hospitalier de Blois, Blois, France (J. Badin, C. Delale, V. Clavier)

**Keywords:** Babesia, *Babesia* crassa, zoonotic disease, immunocompromised patient, PCR, phylogenetic, France, vector-borne diseases, tickborne diseases

## Abstract

Human babesiosis in Europe is caused by multiple zoonotic species. We describe a case in a splenectomized patient, in which a routine *Babesia divergens* PCR result was negative. A universal *Babesia* spp. PCR yielded a positive result and enabled classification of the parasite into the less-described *Babesia crassa*–like complex.

Babesiosis is a widely distributed, tickborne, zoonotic, parasitic disease caused by different species of the apicomplexan genus *Babesia* and occasionally involving human infections (*1*). In its vertebrate host, the parasite undergoes repeated erythrocytic cycles. Clinical manifestations in humans vary widely, ranging from asymptomatic infections to rapidly evolving and sometimes fatal infections. In Europe, symptomatic human cases are infrequently observed and occur mostly in asplenic patients, where infections can rapidly become life-threatening. The most known species in Europe are *Babesia divergens* and *B. venatorum*, which are naturally found in cattle and deer (*2*). In contrast, infections in the United States are predominantly attributed to the rodent parasite species *B. microti* in the Northeast and Midwest and to *B. duncani* on the Pacific Coast and are more frequently described in human cases (*3*). These cases are normally mild to moderate in immunocompetent persons but can be fatal in asplenic patients. 

Reports of *B. microti* in ticks (*4*) and humans in Germany and Poland (*5*,*6*) and *B. divergens* in the United States (*7*) cast doubt on the reliability of these clear-cut geographic patterns. In addition, numerous zoonotic species exist and are occasionally described in human cases (*8*). Given the life-threatening potential of *Babesia* infections, rapid and reliable diagnostic methods are needed. Results of serologic testing are often negative during the acute phase. Moreover, sensitivity and specificity are, especially for nonclassical species, not yet well described. Direct parasite detection is therefore preferable. PCR tests are performed in some specialized laboratories. However, they are usually designed to detect the major species, notably *B. divergens* and *B. microti*. We present a case study that demonstrates the need to develop a consensus for a general molecular means of detecting *Babesia*.

## The Case Report

This case report was approved by the Ethics Committee of Medical Faculty and University Hospital of Strasbourg, France. A 61-year-old man from western France visited the emergency department of a general hospital for elevated fever, dyspnea, and jaundice. The patient had undergone gastric cancer–related gastrectomy and splenectomy 30 years before. He lived in an isolated woodland environment and raised goats. At initial examination, the only clinical abnormality was oliguria with dark urine. A blood test revealed acute renal failure (creatinine 5.6 mg/dL [reference range 0.7–1.3 mg/dL]), anemia (Hb 112 g/L [reference range 130–170 g/L]), and thrombocytopenia (18,000 platelets/μL [reference range 150,000–450,000 platelets/μL]) with hyperbilirubinemia (bilirubin 10.9 mg/dL [reference range <1.2 mg/dL]). 

That night, the patient experienced septic shock and was transferred to an intensive-care unit (ICU). Upon arrival, the patient received fluid challenge associated with vasopressor treatment and broad-spectrum antibiotics (ceftriaxone, metronidazole, and amikacin) for a suspected urinary or biliary infection. A new cellular and biochemical blood examination gave no result because of hemolysis. Twelve hours after ICU admission, the blood sample was again hemolyzed. Microscopic analysis of a blood smear showed intracellular and extracellular parasites suggestive of *Babesia*, demonstrating parasitemia of 14%. A combination treatment with quinine (8 mg/kg/8 h) and dalacine (600 mg/8 h) was started. Antibiotic therapy by ceftriaxone was continued for confirmed urinary sepsis with *Escherichia coli* bacteremia. 

On day 2 after admission, the patient was anuric, and renal replacement therapy was started. On day 4, the patient was put on mechanical ventilation because of septic cardiac failure–induced respiratory failure. That day, a tick was found on the patient. The species remained unknown because the tick was not sent to a laboratory. Lyme serologic testing was requested and returned positive results, so ceftriaxone was administered for 3 weeks and quinine/dalacine for 10 days, yielding a negative parasitemia at the end of treatment. The patient slowly recovered; mechanical ventilation and catecholamines were stopped on day 7, dialysis on day 25. He left the ICU on day 34 and left the hospital on day 70 after regaining normal renal function; he returned home after readaptation on day 117.

**Figure 1:**
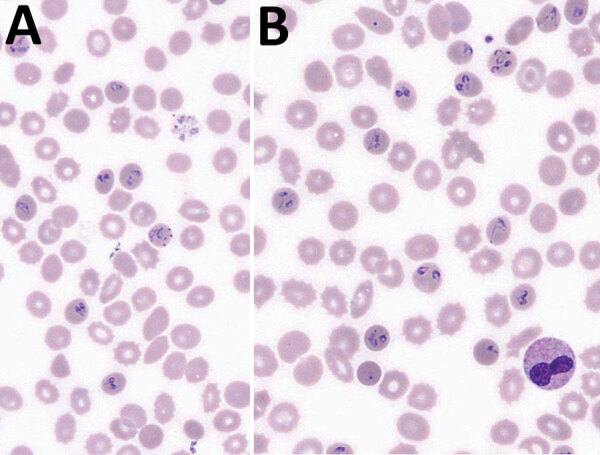
Two representative microscopic fields (original magnification ×1,000) of a May–Grünwald–Giemsa stained blood smear, showing different forms of *Babesia* trophozoites, from a 61-year-old man from western France.

Microscopic examination (Figure 1; Appendix Figure) showed characteristics typically described for *Babesia* trophozoites, including extracellular parasites, abundant binary fission, and absence of schizonts. The observed forms were highly pleomorphic. We observed piriform parasites, resembling *B. divergens*, as well as more round forms, as shown in the original description of *B. crassa* (*9*), but the round forms were not as abundant as usually described. We also observed voluminous forms resembling bandform trophozoites of *Plasmodium malariae.* Although 4 parasites in 1 erythrocyte were frequently observed, the tetrad (Maltese Cross) form, typical for *B. divergens*, was never seen.

**Figure 2:**
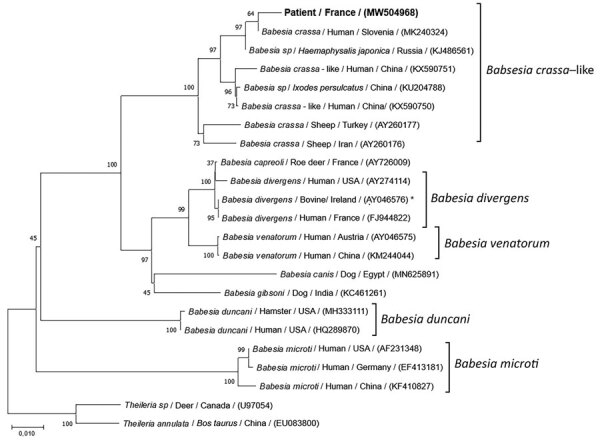
Evolutionary analysis of 18S RNA sequences of *Babesia* from a 61-year-old man from western France and reference sequences. Neighbor-joining tree of 1,000 bootstrap pseudoreplicates with Kimura 2-parameter distances of internal transcribed spacer 1 gene from 18S RNA sequences of the Babesia genus (MEGA X 10.1.8, https://www.megasoftware.net). Bootstrap proportions >50% are indicated. This phylogenetic tree illustrates the relationship between the species infecting this patient (GenBank accession no. MW504968) and the 20 different species of *Babesia* obtained from GenBank. Species, host, origin, and accession number are indicated. *Theileria* spp. was used as outgroup. Scale bar represents 1% of divergence. Asterisk indicates in vitro culture.

We performed our routine PCR tests for *B. divergens* and *B microti*, using LightCycler FastStart DNA Master HybProbe (Roche, https://www.roche.fr) (Appendix). Unexpectedly, both PCRs came back negative. We then applied a universal *Babesia* spp. PCR, targeting a consensus sequence of the internal transcribed spacer 1 gene of the 18S RNA, as previously reported (*10*) (Appendix). Visual inspection of the agarose gel showed a PCR fragment of ≈480 bp. The PCR product was purified and sequenced on both strands by Eurofins Genomics (https://www.eurofins.com). We identified the consensus sequence (GenBank accession no. MW504968) as *Babesia* spp. by using BLAST (https://blast.ncbi.nlm.nih.gov/Blast.cgi). We conducted phylogenetic and molecular evolutionary analyses by using MEGA X 10.1.8 (https://www.megasoftware.net) (*11*). We constructed a phylogenetic tree with corresponding sequences of the *Babesia* genus obtained from GenBank by using the neighbor-joining method with Kimura 2-parameter distances and using *Theileria* spp. as the outgroup (Figure 2). Our sequence aligned with the *B. crassa* complex and specifically with a *B. crassa*–like sequence from Slovenia (GenBank accession no. MK240324) with 99.11% identity.

## Conclusions

Recent serologic and clinical studies suggest that human babesiosis infections are more frequent than expected, especially in Europe, but symptoms are often not recognized as babesiosis (*12*). Microscopic identification of *Babesia* is easily possible in a case-patient with high parasitemia, as in the case we describe. However, in the early phase of infection or in immunocompetent patients, parasitemia often is too low to be detected by routine examination, especially outside specialized laboratories. Therefore, PCR is crucial for reliable diagnosis. The negative result we obtained using our routine PCRs, despite substantial parasitemia, demonstrates once more that unexpected species can be found in human samples and underscores the need to use universal *Babesia* primers and sequencing of amplicons in positive samples, such as the PCR we used to successfully detect the parasites (*10*). Sequencing identified the isolate as being close to *B. crassa*, which was originally described in sheep in Iran (*9*) and was later phylogenetically characterized (*13*). Human infections with *B. crassa*–like parasites, all with mild to moderate clinical symptoms, have been described in China, along with numerous isolations from ticks and sheep (*14*), and from an asplenic patient in Slovenia (*15*), demonstrating the wide geographic distribution of these parasites. Our case proves its presence in France, again in a splenectomized patient, which is probably just the visible part of a wider unnoticed presence in Europe. Detailed microscopic and genetic analysis of more isolates would be useful to better characterize this poorly described complex. Cases of *B. crassa*–like infection can be expected in wildlife throughout the palearctic region and sporadically in humans, especially immunocompromised persons.

In summary, we demonstrate that *Babesia* infections in Europe and elsewhere might implicate species not yet been described in humans, which could lead to false-negative PCR results and delayed treatment of patients at high risk. All facilities performing *Babesia* diagnostic tests should be aware of this possibility and make sure that PCRs are adapted accordingly.

Appendix Additional information about *Babesia crassa*–like human infection indicating need for adapted PCR diagnosis of babesiosis, France.
