# Supplementary material for: Babesia crassa–Like Human Infection Indicating Need for Adapted PCR Diagnosis of Babesiosis, France
Source: Emerg Infect Dis. 2022 Feb;28(2):449–52. doi: 10.3201/eid2802.211596 (PMC8798681; doi:10.3201/eid2802.211596)
Supplement: Appendix — Additional information about Babesia crassa–like human infection indicating need for adapted PCR diagnosis of babesiosis, France. [file 21-1596-Techapp-s1.pdf]

Article DOI: https://doi.org/10.3201/eid5802.211596

Babesia crassa–Like Human Infection Indicating Need for Adapted PCR Diagnosis of Babesiosis, France

Appendix

Protocol of Our Routine PCR

DNA extraction from 100 µL of whole blood was performed according to the "Pathogen universal 200" program on a MagNA Pure 96 Instrument (Roche, Meylan, France) using MagNA Pure 96 DNA and Viral NA Small volume Kit (Roche, Meylan, France), preceded with lysis using 100 µL MagNAPure 96 Bacterial Lysis Buffer and 10 µL Proteinase K (solution containing >600 mAU/ml, QIAGEN, Courtaboeuf, France) according to the manufacturer's instructions. PCR reactions were set up with extracted DNA (2 µL for *B. divergens*, 5 µL for *B. microti*), 1 x LightCycler FastStart DNA Master HybProbe (Roche), 0.9 µM primers (Invitrogen, Courtaboeuf, France) (Bdiv_mic_Fw 5′ACAACGATGAAGGACGCAG3′ for both reactions and either Bdiv_Rv 5′GATCACACGTGGCGATACC3′ for *B. divergens* detection or Bmic_Rv 5′ TCAGCGGATCRTCACATCC3′ for *B. microti* detection) and 0.2 µM probe (Eurogentec, Serain, Belgium) (Bdiv_probe FAM-CGTTTCAGTGAGCCCCCTTTCCT-BHQ1, Bmic_probe YY-AGTGCACCCATTTCAGCGCCT-BHQ1) and 4 mM $MgCl_2$. PCR was performed using a fluorescence detecting temperature cycler (LightCycler 2.0, Roche). The reaction mixture was incubated for 10 min at 95°C, followed by 50 cycles of amplification, 10s at 95°C, 20s at 56°C and 20s at 72°C. The generation of target amplicons was monitored at 530 nm for *B. divergens* or 560 nm for *B. microti*. Samples were considered positive when their fluorescence exceeded background fluorescence, confirmed by visual inspection of the graphical plot generated by the instrument.

Universal *Babesia* spp. PCR

The PCR was performed as described in (*1*), with modifications. Briefly, 5 µl of template DNA were added to the reaction mix containing 0.5 µM of each primer, 1.5mM MgCl2, 0.2mM dNTP, 1unit of HotStarTaq Plus DNA Polymerase and 10x associate buffer (Qiagen, Courtaboeuf, France) in a final volume of 25µl. A touchdown protocol of 40 cycles was conducted: 30s at 95°C, 30s at 55°C with a decrement of 0.5°C per cycle, 30s at 72°C. PCR products were separated on an agarose gel and visually inspected.

Reference

1. Casati S, Sager H, Gern L, Piffaretti J-C. Presence of potentially pathogenic *Babesia* sp. for human in *Ixodes ricinus* in Switzerland. Ann Agric Environ Med. 2006;13:65–70. PubMed

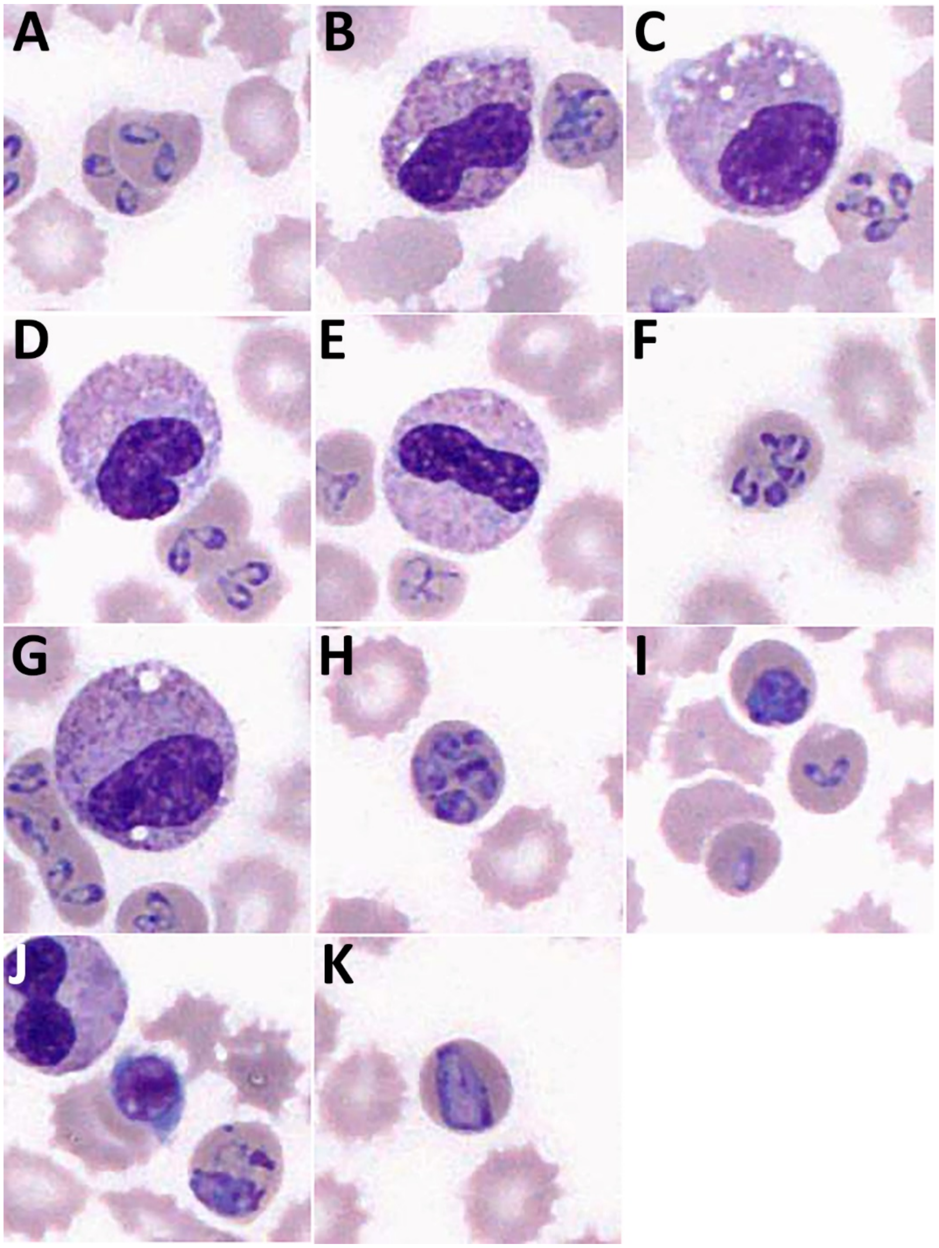

Appendix Figure. Different forms of *Babesia* spp. trophozoites observed in our patient. May–Grünwald–Giemsa stained blood smear (original magnification ×1,000).
